# Suppression of spin rectification effects in spin pumping experiments

**DOI:** 10.1038/s41598-021-04319-z

**Published:** 2022-01-07

**Authors:** Sergi Martin-Rio, Carlos Frontera, Alberto Pomar, Lluis Balcells, Benjamin Martinez

**Affiliations:** Instituto de Ciencia de Materiales de Barcelona, ICMAB-CSIC, Campus UAB, 08193 Bellaterra, Spain

**Keywords:** Materials science, Condensed-matter physics, Spintronics

## Abstract

Spin pumping (SP) is a well-established method to generate pure spin currents allowing efficient spin injection into metals and semiconductors avoiding the problem of impedance mismatch. However, to disentangle pure spin currents from parasitic effects due to spin rectification effects (SRE) is a difficult task that is seriously hampering further developments. Here we propose a simple method that allows suppressing SRE contribution to inverse spin Hall effect (ISHE) voltage signal avoiding long and tedious angle-dependent measurements. We show an experimental study in the well-known Py/Pt system by using a coplanar waveguide (CPW). Results obtained demonstrate that the sign and size of the measured transverse voltage signal depends on the width of the sample along the CPW active line. A progressive reduction of this width evidences that SRE contribution to the measured transverse voltage signal becomes negligibly small for sample width below 200 μm. A numerical solution of the Maxwell equations in the CPW-sample setup, by using the Landau-Lifshitz equation with the Gilbert damping term (LLG) as the constitutive equation of the media, and with the proper set of boundary conditions, confirms the obtained experimental results.

## Introduction

Spin pumping (SP) is a well-established method to generate pure spin currents i.e., a pure spin current is emitted at the interface between a ferromagnet (FM) with a precessing magnetization and a normal-metal (NM)^1–3^. SP allows efficient spin injection into metals and semiconductors avoiding the problem of impedance mismatch^4^. Since SP implies the opening of a new way for dissipating angular momentum it is easily detectable by the increase of the magnetic damping, *α*, i.e. through the increase of the ferromagnetic resonance (FMR) linewidth^5^. However, the effective spin current injected into the NM may be substantially reduced, or even fully suppressed, due to interfacial loss of spin coherence^6^, thus a more reliable proof of effective spin injection into the NM is obtained through inverse spin Hall effect (ISHE), i.e. a pure spin current generates a charge current due to the spin–orbit interaction^7^. The interconversion between charge current and spin current is completed by spin Hall effect (SHE) that is the reciprocal effect to ISHE^8^. ISHE enables an electrical detection of a pure spin current according to the expression^7^: ***J***_***C***_ = (2*q*_*e*_/*ħ*)*θ*_*SH*_***J***_***S***_ × ***σ***, being ***J***_***S***_ the spin current, *ħ* the reduced Planck’s constant, *q*_*e*_ the electron charge, ***σ*** is the spin polarization vector and *θ*_*SH*_ is the Hall angle, which quantifies the conversion efficiency between charge and spin currents. However, the voltage signal detected in FMR experiments on metallic FM/NM devices may also have contributions coming from spin rectification effects (SRE)^9^. SRE appear due to the nonlinear synchronous coupling between an oscillating eddy current, induced by the magnetic field of the microwave, and an oscillating resistance in magnetic materials giving place to the appearance of a dc voltage/current^9^. Therefore, SRE enable the study of magnetization dynamics using electrical measurements in a broad range of magnetic materials including metals, semiconductors and insulators. SRE are also behind the recent development of techniques such as Spin Torque ferromagnetic resonance (ST-FMR) that allow a complete characterization of dynamic magnetic properties, such as *α* and *θ*_*SH*_^10,11^. Thermoelectric effects, i.e. Seebeck and Nernst effects, may also contribute to generate an electromotive force^12^. In a FM/NM heterostructure SRE are typically integrated by anisotropic magnetoresistance (AMR) and anomalous Hall effect (AHE) due to spin–orbit coupling in the FM layer^13^. Separating the voltage generated by ISHE from parasitic contributions due to SRE in spin pumping experiments is a complicated work that has been tackled by several authors in the past few years but is still not fully resolved. Pioneer methods for separating ISHE and SRE were based on line shape separation assuming that the symmetric contribution is coming uniquely from ISHE^14^. Nevertheless, SRE also contribute to the symmetric part of the transverse voltage signal^15^. A more elaborated analysis of line shape was proposed by Mosendz et al.^16,17^. However, its applicability is severely limited due to the specific requirements of the experimental setup and the experimental conditions. A separation method based on the different angular and field symmetries of ISHE and SRE was proposed by Bai et al.^18^ but a very precise control of the field orientation and a high-power microwave source are required, thus limiting its practical application. Other separation methods rely on the different magnetic field orientation dependence of ISHE and SRE signals^19–22^ however, they require a long and tedious measurement process. The different dependencies on the thicknesses of the FM and NM layers has been used to disentangle ISHE and SRE contribution to the measured voltage signal^23^. Obviously, this method requires a large number of samples and measurements, as well as a proper account of the thickness dependence of different parameters, such as the resistivity of the FM or the spin mixing conductance. Separation of both signals by using the different behavior of ISHE and SRE under the inversion of the direction of spin injection has also been reported^24,25^. The corresponding signals are obtained simply by adding and subtracting the voltage signals measured by inverting the spin injection direction. However, reversing the stacking order in the bilayer may severely affect the quality of the interfaces and therefore, modify the effective spin injection. Thus, the direct addition/subtraction procedure may be affected by this experimental error. An alternative procedure, consisting of flipping the whole sample covered by a pristine substrate on top, to ensure as much as possible similar experimental conditions in both measurements, has also been proposed^23^. Nevertheless, this requires to make substrates on both sides thinner to guarantee a good signal to noise ratio.

In this work we propose a new method to suppress the SRE contribution leading to a straightforward measurement of the ISHE transverse voltage signal in SP experiments in FM/NM bilayers. This method can be easily implemented and allows a full suppression of the SRE signal in SP experiments in coplanar waveguide (CPW) and microstrip experimental setups.

## Results

FMR and transverse voltage signals measurements have been determined simultaneously. At the resonance frequency an absorption Lorentzian-shaped peak appears in the transmission coefficient of the CPW, *S*, whose derivative is described by the expression^26^:

1 $$\frac{dS}{dH}=\frac{d}{dH}\left\{\frac{{k}_{S}\Delta {H}^{2}+{k}_{AS}\Delta H\left(H-{H}_{res}\right)}{\Delta {H}^{2}+4{\left(H-{H}_{res}\right)}^{2}}\right\}$$

being *k*_S_ and *k*_AS_ the symmetric and antisymmetric FMR constants and *H*_*res*_ and Δ*H* are the resonance field and linewidth, respectively. These two parameters are related to the magnetic features of the samples through the Kittel equations^27^:

2 $$f={\mu }_{0}\frac{\gamma }{2\pi }\sqrt{\left({H}_{res}+{H}_{k}\right)\left({H}_{res}+{H}_{k}+{M}_{s}\right)}$$

3 $$\Delta H=\Delta H\left(0\right)+4\pi \frac{\alpha }{\gamma }f$$

where *f* is the resonant frequency, γ = *g*μ_*B*_/ħ is the gyromagnetic ratio (in units of GHz/T), μ_0_ is the vacuum permeability, *H*_*res*_ and *H*_*k*_ are the resonant and anisotropy fields respectively (*H*_*k*_ is nearly zero in magnetically isotropic Py films), *M*_S_ is the saturation magnetization of the Py film, Δ*H*(0) is the so-called inhomogeneous line broadening and α is the Gilbert damping constant^28–30^.

**Figure 1:**
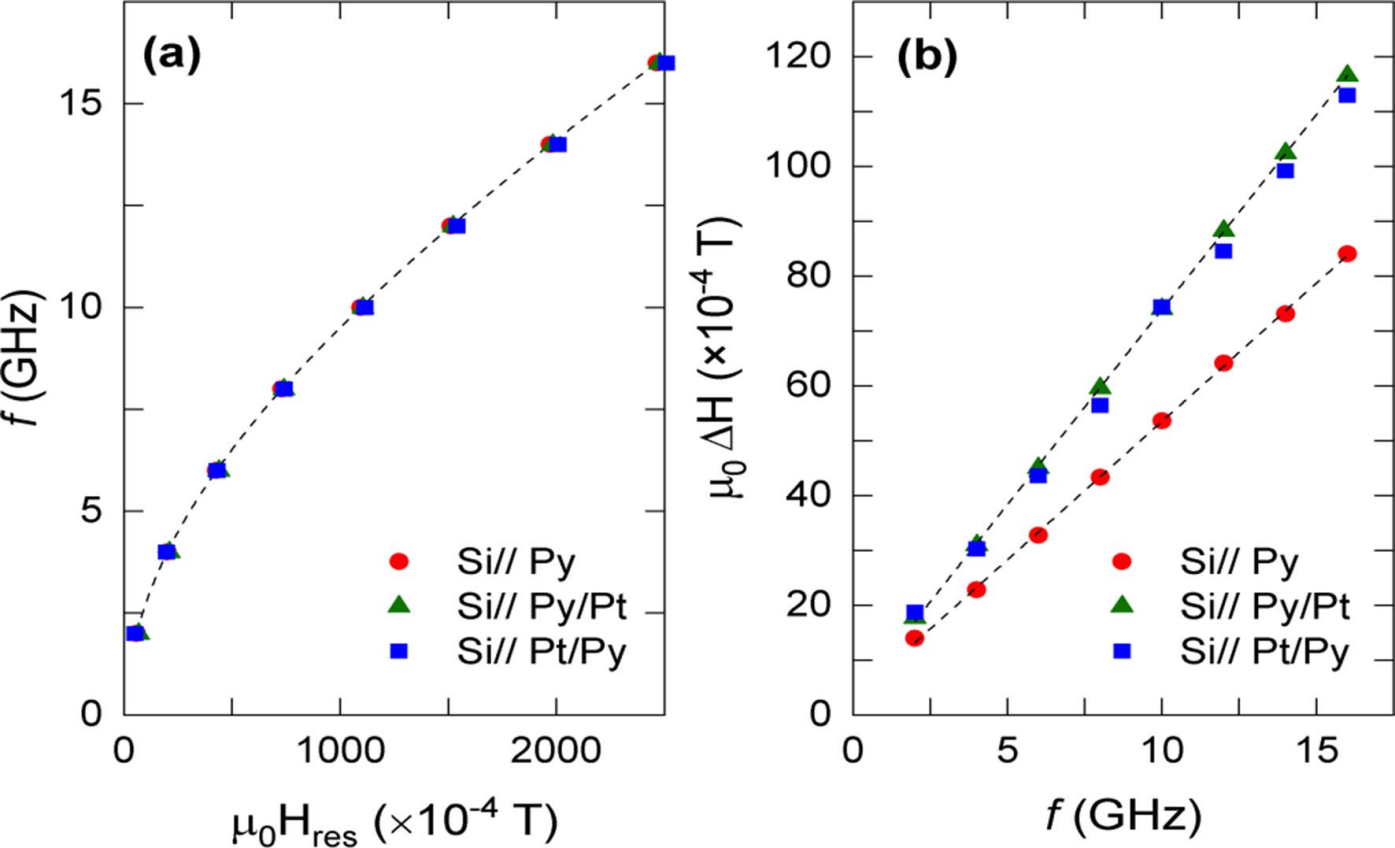
Representative curves showing the dependence of the resonant frequency on the magnetic field for the three sets of films at room temperature (**a**). Frequency dependence of the absorption linewidth at room temperature for a Py layer compared with that of Py/Pt and Pt/Py bilayers. The increase of the damping in the bilayers is clearly shown (**b**). Sample dimensions: 5 × 5 mm^2^.

The values obtained for both *M*_*S*_ and α in Py alone films are in good agreement with values previously reported (see Supplementary Information, Table S1)^15,31,32^. On the other hand, values of Δ*H*(0) are low, as expected for a magnetically and structurally homogeneous system. A substantial increase of the effective damping, α_eff_, is detected in Py/Pt bilayer samples which would be indicative of the existence of spin injection (see Fig. 1). The SP process can, therefore, be viewed as an extrinsic contribution to the Gilbert damping α_eff._ = α + α_sp_, whose value can be estimated from the increase of the FMR linewidth, *ΔH*, in samples with and without Pt layer^6,15^. The enhancement of the magnetic damping allows also determining the effective spin-mixing conductance, ${\text{g}}_{ef}^{\uparrow \downarrow }$^6,15,33^.

4 $${\text{g}}_{{ef}}^{{ \uparrow \downarrow }} = \frac{{4\pi M_{S} t_{{FM}} }}{{\gamma \hbar }}\Delta \alpha {\text{ = }}\frac{{{\text{4}}\pi {\text{M}}_{{\text{S}}} {\text{t}}_{{{\text{FM}}}} }}{{\gamma \hbar }}\left( {\alpha _{{\frac{{{\text{Py}}}}{{{\text{Pt}}}}}} - \alpha _{{{\text{Py}}}} } \right)$$

Being *t*_*FM*_ the thickness of the FM layer and α_Py_ and α_Py/Pt_ the damping of the Py layer and of the Py/Pt bilayer respectively. The value obtained in our samples (*t*_*Py*_ ~ 16 nm) is ${\text{g}}_{ef}^{\uparrow \downarrow }$~ (2.31 ± 0.27) × 10^19^ m ^−2^, in good agreement with previous values reported for Py/Pt^15,34,35^.

The effective spin injection into the Pt layer is detected through the transverse voltage signal generated by ISHE. Other potential sources, i.e. thermoelectric effects, are discarded since the temperature increase at resonance is about few hundreds of mK, even at large RF power^36^, thus their contribution to the final voltage signal is irrelevant. The voltage signal is generated by the same magnetization dynamics that governs FMR, thus the line shape of the voltage curves should be a Lorentzian^9^:

5 $$V=\frac{{V}_{S}\Delta {H}^{2}+{V}_{AS}\Delta H\left(H-{H}_{res}\right)}{\Delta {H}^{2}+4{\left(H-{H}_{res}\right)}^{2}}$$

where V_S_ and V_AS_ correspond to the symmetric and antisymmetric voltage amplitudes, respectively. The signal due to ISHE should only depend on the cone angle of the magnetization precession being, therefore, fully symmetric. However, SRE also contribute to the symmetric part of the experimental signal complicating the separation of both signals.

**Figure 2:**
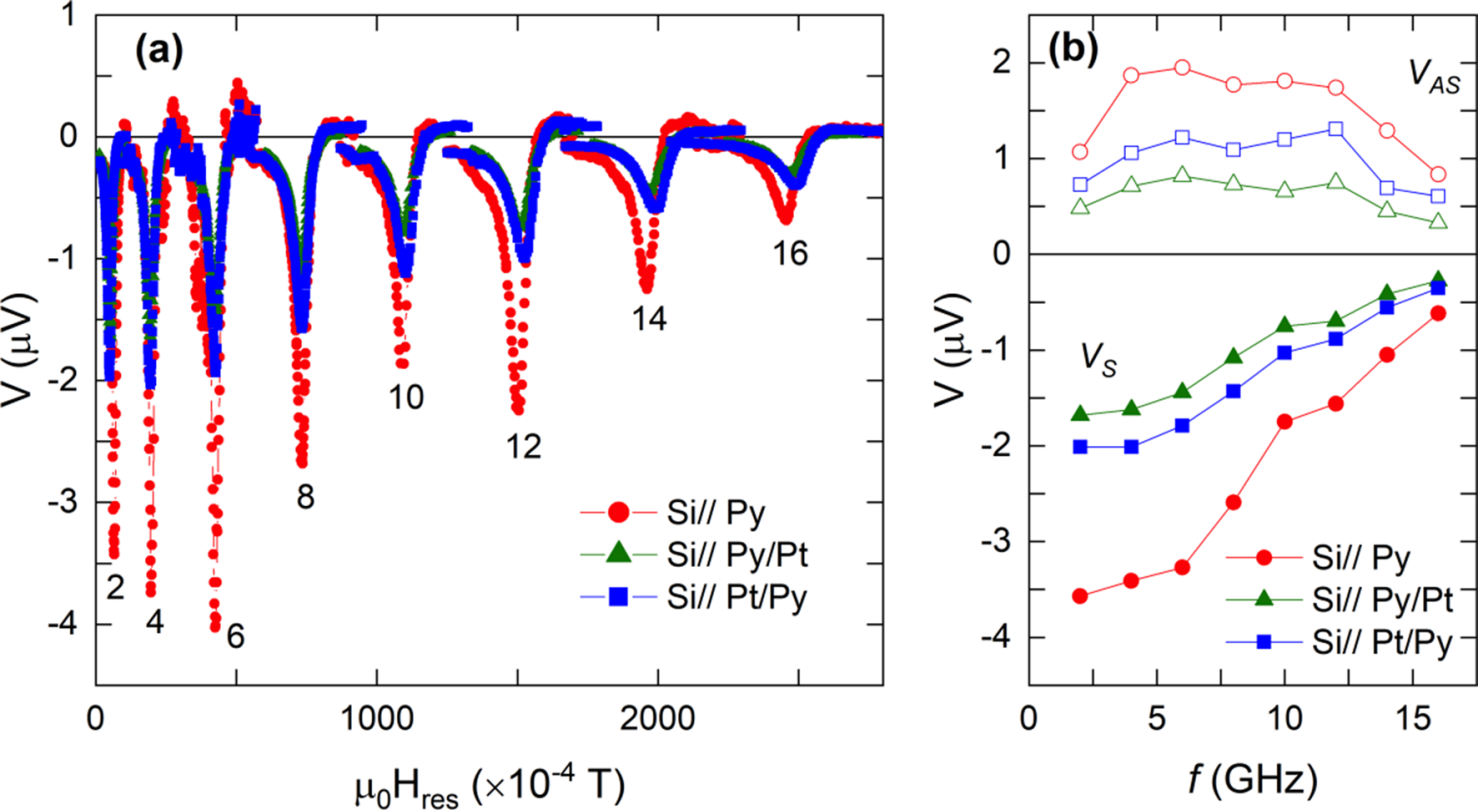
(**a**) Representative transverse voltage signal for the three set of samples as a function of the applied magnetic field for different frequencies (GHz) as indicated in the figure. (**b**) Amplitude of the symmetric (full symbols) and antisymmetric (open symbols) components of the transverse voltage signal of each curve as a function of frequency. Sample dimensions: 5 × 5 mm^2^.

The voltage signal corresponding to different 5 × 5 mm^2^ samples (see Table S1), as a function of the frequency, is shown in Fig. 2a. All the curves show the same shape as a function of the applied magnetic field. However, they have different intensities due to small differences in the absorption of the ℎ_*rf*_ field, higher resistivity of Py compared to Pt and/or the contribution due to ISHE.

It is also worth noticing that the measured voltage signal is negative. However, considering the electrical connections in our setup (see Supplementary Information. Fig. S1b), ISHE voltage signal is expected to be positive in the case of Si//Py/Pt stacking since both Pt and Py have a positive Hall angle^6,12^. Voltage curves in Fig. 2a have been analyzed by using Eq. (5) and the voltage amplitudes corresponding to the symmetric and antisymmetric contributions as a function of frequency are depicted in Fig. 2b. From this figure it is evident that the relative magnitude of the symmetric and antisymmetric voltage amplitudes is very similar for each sample and frequency. This result evidences that in samples with width, *W*, along the CPW of *W* = 5 mm, SRE are the dominant contribution to the measured transverse voltage signal. The origin of the SRE (occurring in the Py film) is the inductive coupling between the sample and the CPW signal line, which creates an eddy current travelling in the opposite direction of the CPW signal line current^25,37^. Therefore, it may be reduced by laminating the sample in the direction of the induced current. According to this, at sufficiently short sample width, *W,* along the CPW signal line the circulating eddy current should be almost zero and therefore, SRE should vanish while ISHE voltage signal should be almost constant.

The dependence of the transverse voltage signal on *W* was measured in the three sets of samples. For that purpose, samples with different values of *W*, ranging from 2 mm to 20 μm, have been analyzed (see Table S2 in Supplementary information).

It is important to notice that all samples, irrespective to *W*, share the same magnetic properties with the 5 × 5 mm^2^ original films (see Table S1). The difference in the Gilbert damping values of Py and Py/Pt bilayers is also maintained. This indicates that, from the magnetic point of view, Py films are not affected by the patterning process.

**Figure 3:**
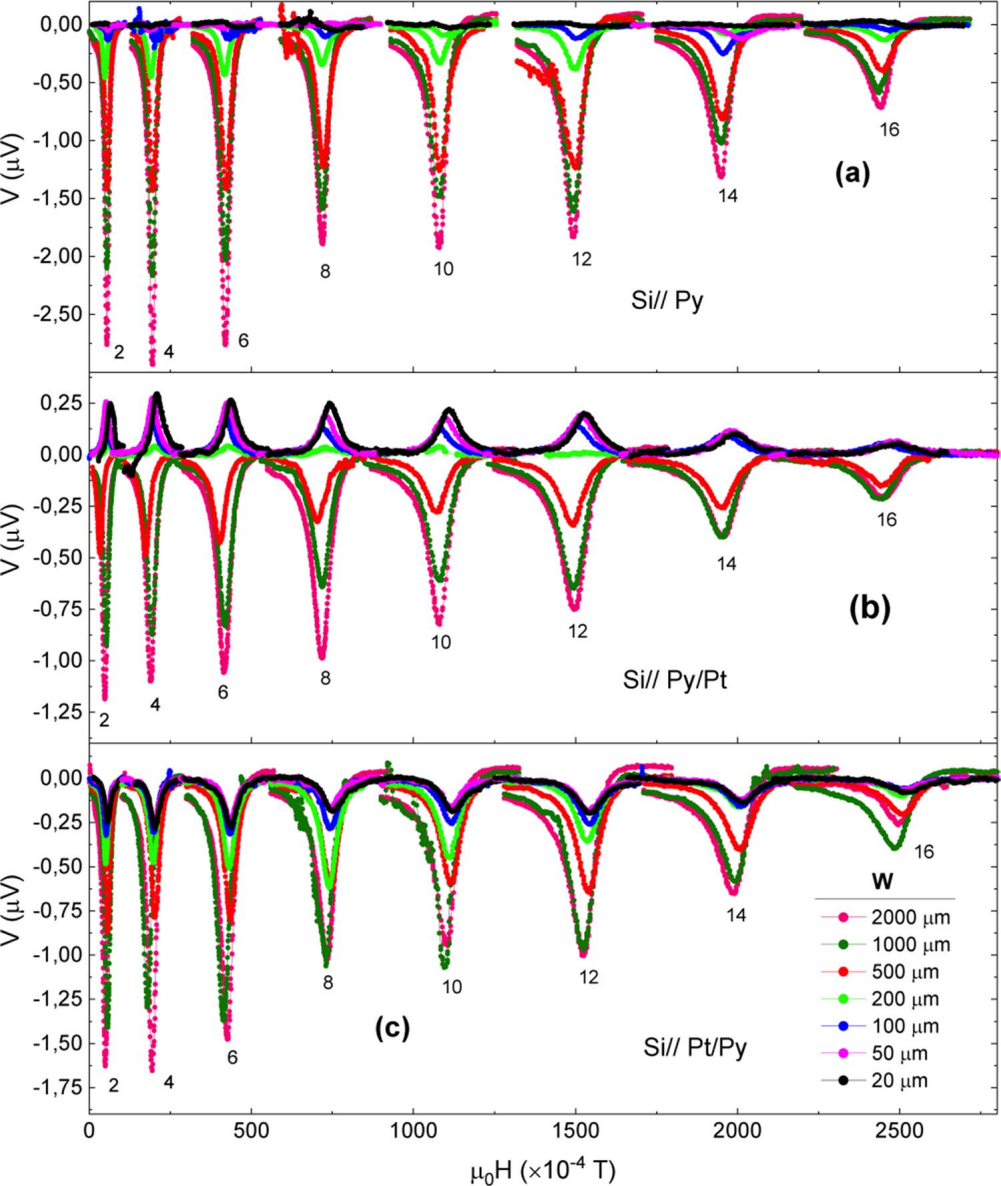
Transverse voltage signal in Si//Py (**a**), Si//Py/Pt (**b**) and Si//Pt/Py (**c**) films with different width, W, as a function of the magnetic field for different frequencies (GHz) as indicated in the figure.

**Figure 4:**
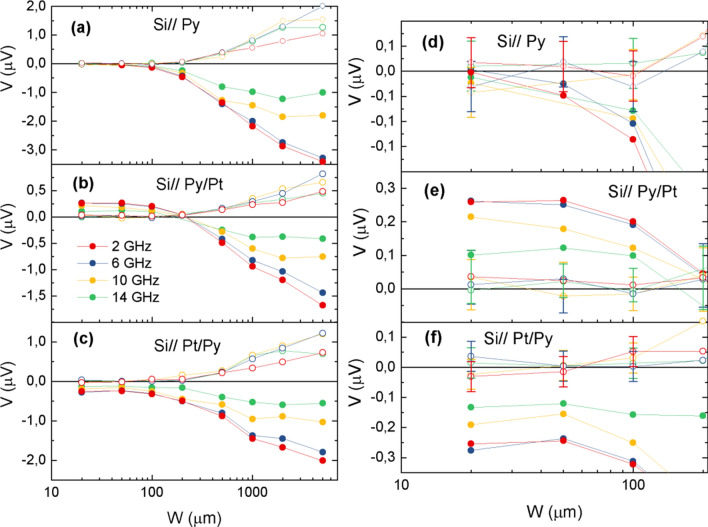
Left: Sample width dependence of the symmetric (full symbols) and antisymmetric (open symbols) components of the transverse voltage signal at different frequencies for Si//Py (**a**), Si//Py/Pt (**b**) and Si//Pt/Py (**c**) films. Right: Zoom of V values close to zero for Si//Py (**d**), Si//Py/Pt (**e**) and Si//Pt/Py (**f**) films.

As shown in Fig. 3a the transverse voltage signal measured in Py samples is always of negative sign irrespective to *W* and its amplitude decreases with decreasing *W*. Moreover, the amplitude of both the symmetric and antisymmetric components also decreases with increasing frequency (see Fig. 4a). It is also worth mentioning that both the symmetric and antisymmetric voltage amplitudes become zero at sufficiently small width, i.e. *W* ≤ 100 μm (see detail in Fig. 4d).

In the case of Py/Pt bilayers the first remarkable feature is that the sign of the voltage signal changes and becomes positive for *W* below about 200 μm (see Fig. 3b). As in the case of Py alone samples, the amplitude of the voltage signal decreases with decreasing *W* above 200 μm. However, for *W* below 200 μm the amplitude of the voltage signal becomes almost constant and fully symmetric (see Fig. 4b,e).

Finally, the stacking order of Py and Pt was inverted to take advantage of the different behavior of ISHE and SRE under the inversion of the spin injection direction^22,23^.

In Pt/Py bilayers the transverse voltage signal at resonance is always negative (see Fig. 3c) irrespective of *W*. However, unlike the case of Py alone samples, a closer look at their symmetric and antisymmetric voltage components reveals that while the amplitude of the antisymmetric component goes to zero with decreasing *W*, the amplitude of the symmetric component remains at a negative value (see Fig. 4c,f). Thus, results for the smaller *W* values are a mirror image of those obtained in the Si// Py/Pt samples, as expected for ISHE considering the inversion of the spin injection direction.

In all the cases it is observed that the intensity of the voltage signal slightly decreases on increasing the frequency, which is contrary to the expected behavior since, in principle, the spin current generated by SP should be proportional to the precession frequency, *f*^3^. However, a slight decrease is observed due to the compensation between the magnetization-precession frequency and the spin current generated by a cycle of the precession, due to their different frequency dependencies^3,38^.

## Discussion

Considering that SRE are generated by a microwave eddy current, at sufficiently short *W* the current should be very small ($\overrightarrow{j}\approx 0$). Therefore, SRE should vanish while ISHE voltage signal should be almost constant since it is not affected by the absence of $\overrightarrow{\text{j}}$ and no dependence on *W* should be observed. As a consequence, below a certain threshold value of *W* (about 200 μm) the contribution of SRE to the transverse voltage signal should be almost zero, as effectively observed in the case of Py alone samples (see Fig. 4a). It is worth noting that contributions to the transverse voltage signal due to self-induced charge current in the Py layer may also exist^39–42^. However, studies of the temperature dependence of the self-induced transverse voltage in Py demonstrate that spin-charge conversion efficiency at room temperature is very low^39^, in agreement with our observation of almost zero transverse voltage signal once SRE contributions have been suppressed. In the Si//Py/Pt samples for large values of *W* the measured voltage signal is: i) negative; but, considering the electrical connections in our experimental setup, ISHE voltage signal should be positive; and ii) has a decreasing amplitude as *W* is reduced (see Figs. 3b and 4b). This behavior is similar to that observed in the Py alone samples and indicates that the measured voltage signal is dominated by the SRE contribution. When the *W* threshold value is approached the SRE contribution almost disappears and the measured voltage signal becomes positive and with a *W*-independent amplitude, thus indicating that only ISHE signal is contributing to the measured voltage signal (see Fig. 4b). In fact, it is observed that the line-shape corresponding to negative voltage values has both symmetric and antisymmetric contributions, while the line-shape associated to positive voltage signals is fully symmetric (see Fig. 4b), thus making evident that they are originated by ISHE.

**Figure 5:**
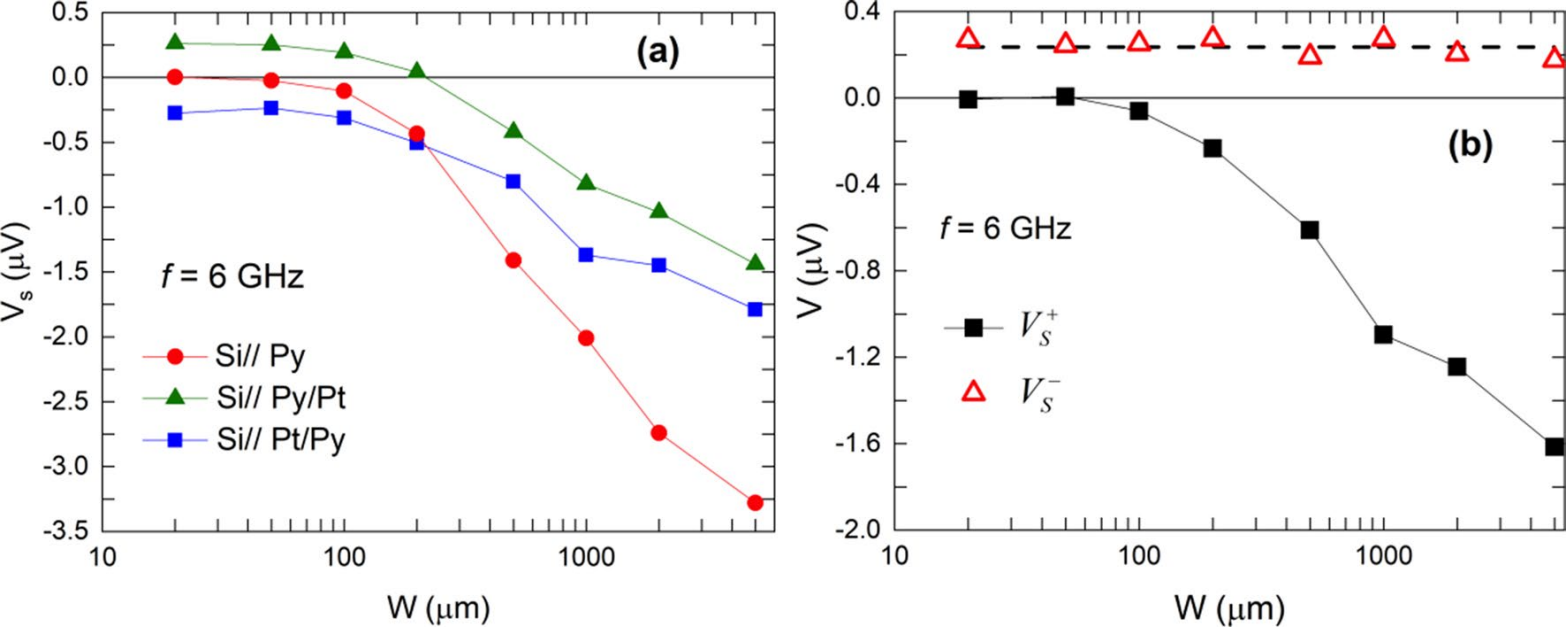
(**a**) Dependence of the amplitude of the symmetric component of the transverse voltage signal of each sample set at room temperature as a function of sample width, W. (**b**) Half-sum (V_S_^+^) and half-difference (V_S_^–^) of the symmetric voltage amplitudes of the Si//Py/Pt and Si//Pt/Py samples at 4 GHz and room temperature. Error bars have, approximately, the size of the symbols.

In samples with inverted stacking order, i.e. Si//Pt/Py no change of sign in the measured transverse voltage signal was observed (see Fig. 3c). This contradicts the expected change in sign due to the inverted spin injection direction, and is due to the fact that SRE do not depend on the spin injection direction while ISHE is an odd function of it^22,23^, and in this configuration SRE and ISHE signals have the same sign. Our picture predicts that, below a certain threshold value of *W,* the amplitude of the signal should be almost constant, since SRE contributions should be zero and ISHE contribution does not depend on *W,* as it is in fact observed (see Fig. 4c). The spectral line-shape analysis confirms that below the threshold value the measured voltage signal is fully symmetric, and does not depend on *W*, while above it both symmetric and antisymmetric contributions are present (see Fig. 4c). Figure 5 shows the dependence of the symmetric component of the voltage amplitude as a function of the sample width, *W,* at a given frequency. The figure clearly illustrates that in Py alone samples the measured transverse voltage signal progressively goes to zero as *W* decreases. However, in both Py/Pt and Pt/Py bilayer systems the symmetric component of the signal saturates at a positive and negative value respectively (according to the expected sign of ISHE), and has almost the same absolute value. This indicates that once SRE effects are suppressed the remaining measured signal corresponds to ISHE. Therefore, since the inversion of the spin injection direction must change the sign of the ISHE signal, while that of the SRE contribution remains the same, half the subtraction of both signals should give the value of the ISHE signal.

In Fig. 5b the half-sum (V_S_^+^) and half-difference (V_S_^-^), defined as:${V}_{s}^{\pm }=\left(1/2\right)\left({V}_{s}^{Py/Pt}\pm {V}_{s}^{Pt/Py}\right)$, of the bilayer symmetric voltage amplitude with respect to *W* are depicted. As expected, a constant value in the half-difference is observed indicating the value of the ISHE component. On the other hand, the half-sum should decrease and go to zero as *W* decreases, since SRE contribution should be progressively reduced, as effectively shown in the picture. The *W* threshold value does not depend on the width of the active line of the CPW neither on the separation of the electrical contacts, provide they are far apart from the CPW active line were excitation of the magnetization takes place. However, it may be slightly dependent on the resistivity of the FM material, so this threshold value may be smaller in a FM material with higher conductivity.

From the values of the ISHE voltage signal, and assuming a *λ*_*S*_≈ 8 nm (according to the resistivities of the Pt layer, namely ρ ~ 10 μΩ cm), a value of the Hall angle, *θ*_*SH*_ ~ 0.015 ± 0.005 is derived. This value is in the range of low values reported in the literature. However, it should be noted that there is a broad range of values of Pt conductivity and, therefore, of the spin diffusion length, *λ*_*S*_, and a clear correlation between *θ*_*SH*_ and *λ*_*S*_ has been observed^43^. Taking into account this relation the value of *θ*_*SH*_ derived in this work is comparable to values reported in systems with similar resistivities of the Pt layer. (See Ref.^43^ and references therein).

**Figure 6:**
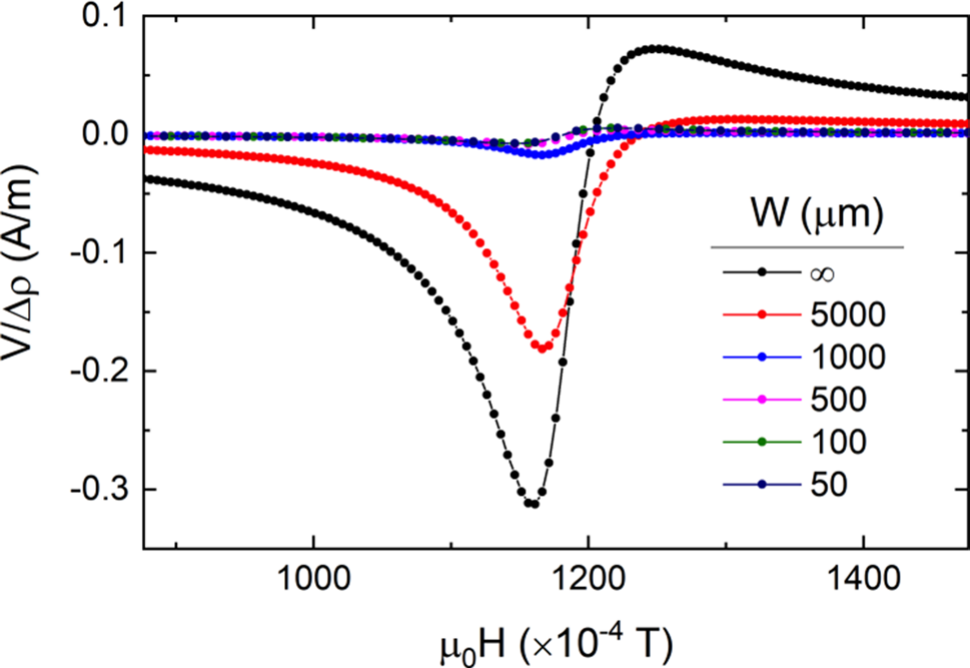
Sample width, W, dependence of the AMR pre-factor plotted according to the sign convention used for experimental measures. Parameters used for the calculations are detailed in the Supplementary information.

To gain a deeper insight into the behavior of the induced current circulating through the Py layer a numerical study of the SRE on Si// Py/Pt bilayers in a CPW experimental setup has been performed. The time dependence of the magnetic field inside the sample induces a time dependent current density $\overrightarrow{j}$ as described by Maxwell’s equations. Riet and Roozeboom^44^ use a quite crude approximation in which the magnetic field inside the ferromagnet is assumed to have only one component (contained in the film plane). In our case, in order to make a little bit more realistic calculations, the methodology presented in the series of papers by Kostylev et al. has been used (see Supplementary information)^24,25,45,46^. The main results derived from the numerical solution are that the dominant effect contributing to SRE is the AMR term. Moreover, the AMR term has both symmetric and antisymmetric components being dominant the symmetric component (see Fig. S8). Results obtained also show that the sign of the SRE voltage signal and that of ISHE voltage signal are opposed for the Si//Py/Pt stacking. Additionally, our estimation of the finite size effects along the CPW direction shows that the voltage contribution to the measured transverse voltage signal progressively decreases as *W* is reduced (see Fig. 6) in agreement with our experimental results.

## Conclusions

We have designed a strategy to suppress SRE contribution to the transverse voltage signal measured in SP experiments in FM/NM bilayers. SRE are generated by a microwave eddy current circulating through the sample due to the inductive coupling between the sample and the signal line of the CPW. Thus, a way to reduce SRE is reducing this circulating current. For that purpose, we have reduced the width, *W*, of the sample along the CPW signal line. According to this, at sufficiently short *W* the circulating current should be very small while ISHE voltage signal should be almost constant since it is not affected by the absence of the induced current. Thus, the contribution of SRE to the measured voltage signal is suppressed. To demonstrate this a careful study of the SP process in three sets of samples (Si//Py, Si//Py/Pt and Si//Pt/Py) as a function of *W* has been performed at room temperature. It is shown that, in the case of Py alone samples, the measured transverse voltage signal is generated by SRE and progressively decreases as *W* is reduced and becomes vanishing small below a threshold value of approximately 200 μm. In the case of the Py/Pt system, the measured transverse voltage signal progressively decreases as *W* decreases, changes sign and saturates at a positive value for *W* values below approximately 200 μm. When the same measurements are performed in a sample with inverted stacking order, i.e. Pt/Py, the measured transverse voltage signal progressively decreases as *W* decreases, but in this case, there is no change of sign and the signal saturates at a negative value, being the absolute value of the signal similar to that measured in the Py/Pt sample. These results demonstrate that SRE in SP experiments, using a CPW or microstrip experimental setup, can be fully suppressed by reducing *W*. An analysis of the rectifications effects in a FM/NM bilayer system on top of a CPW by finding a numerical solution of the Maxwell’s equations, using the Landau-Lifshitz equation with the Gilbert damping term (LLG) as the constitutive equation of the media, also confirms that the expected contribution of the SRE: (i) has symmetric and antisymmetric contributions; (ii) is against ISHE in Py/Pt stacking sequence; and (iii) strongly diminishes as *W* is reduced.

## Materials and methods

Three different sets of samples, namely, i) Si// Py, ii) Si// Py/Pt, and iii) Si// Pt/Py have been prepared by using DC magnetron sputtering technique. All samples have been grown in situ on top of Si (100) substrates, that have a native SiO_2_ passivation layer, at room temperature and using 2.7 mTorr (Py) and 5 mTorr (Pt) of Ar-H_2_ working gas pressure. The thickness of the Py (Ni 80%-Fe 20%) and Pt layers, determined by X-ray reflectometry, (measured using a Siemens D5000 diffractometer) was estimated to be 15–16 nm and 5 nm, respectively for all samples. UV photolithography was used for the patterning of the single-striped and multiple stripes (fringed) samples in a 10,000 class cleanroom (ISO7). Using a combination of lithographic and ion milling techniques 5 × 5 mm^2^ thin films have been transformed into 5 × *W* mm^2^ stripe-shaped samples, where *W* is the width of the stripe. Samples with different values of *W*, ranging from 5 mm to 50 μm, have been prepared, while for smaller values of *W* (20 µm) a fringed pattern was used. Single striped samples were 5 mm long, while fringed samples were only 2 mm long. The use of fringed patterns instead of a single strip for *W* = 20 µm is justified by the fact that for values of *W* below approximately 100 µm FMR measurements are too noisy, making it difficult to determine the fitting parameters, $\Delta H$ and ${H}_{res}$. Additionally, the global voltage drop across the fringed pattern is the average voltage drop across *each* individual strip (see supplementary information).

The static magnetic properties of the different samples were studied by using a SQUID magnetometer (MPMS-X7 by Quantum Design). Py layers exhibit coercive fields (μ_0_H_C_) on the order of 10^–4^ T and a saturation magnetization around 0.88 T (See Supplementary material) in good agreement with results reported in the literature. The dynamic magnetic properties were studied by means of a ferromagnetic resonance spectrometer (FMR) made of a broadband coplanar waveguide (CPW) (NanOsc), inserted in a physical properties measurements system (PPMS by Quantum Design) using a lock-in differential detection method. The transverse voltage signal across the sample was measured using a Keithley 2182A nanovoltmeter. Sample is located upside-down and connections are made at both sides by pins already mounted on the sample holder (See Fig. S1b). Electrical contacts (Au) have been deposited Ex situ by dc-magnetron sputtering. Finally, UV photolithography was used for the patterning of the single-striped samples and multiple stripes samples (fringed patterned). A schematic representation of both systems is shown in Fig. S1a.

## Supplementary Information

Supplementary Information.
