# Supplementary material for: Suppression of spin rectification effects in spin pumping experiments
Source: Sci Rep. 2022 Jan 7;12:224. doi: 10.1038/s41598-021-04319-z (PMC8742073; doi:10.1038/s41598-021-04319-z)
Supplement: Supplementary file 1 — Supplementary Information. [file 41598_2021_4319_MOESM1_ESM.pdf]

Supplementary Information

SUPRESSION OF SPIN RECTIFICATION EFFECTS IN SPIN PUMPING EXPERIMENTS

Sergi Martin-Rio, Carlos Frontera, Alberto Pomar,

Lluis Balcells and Benjamin Martinez

Instituto de Ciencia de Materiales de Barcelona, ICMAB-CSIC, Campus UAB, 08193 Bellaterra, Spain

Corresponding author:

Prof. Benjamin Martinez.

Instituto de Ciencia de Materiales de Barcelona, ICMAB-CSIC, Campus UAB, 08193 Bellaterra, Spain

e-mail: ben.martinez@icmab.es

Phone: +34 93 580 18 53

Table S1. Magnetic parameters for the different sets of 5×5 mm^2 samples as determined by FMR. (average ± standard deviation). The thickness of the Py and Pt layers was estimated to be 15-16 nm and 5 nm, respectively for all samples.

Sample	# samples measured	$\mu_0 M_s$ (T)	$\gamma/2\pi$ (GHz/T)	$\alpha_{eff} \times 10^{-4}$	$\mu_0\Delta H(0)$ ($\times 10^{-4}$ T)
Si// Py	4	0.94 ± 0.01	29.68 ± 0.04	74.25 ± 0.85	2.98 ± 0.37
Si// Py/Pt	4	0.94 ± 0.01	29.69 ± 0.03	107.50 ± 1.15	2.83 ± 0.07
Si// Pt/Py	2	0.92 ± 0.01	29.60 ± 0.07	100.95 ± 0.15	2.43 ± 0.31

Table S2. Magnetic parameters for the different sets of samples with varying width (*W*) obtained from FMR fits. The thickness of the Py and Pt layers was estimated to be 15-16 nm and 5 nm, respectively for all samples.

Sample	Width *W* (μm)	$\mu_0 M_s$ (T)	$\gamma/2\pi$ (GHz/T)	$\alpha_{eff} \times 10^{-4}$	$\mu_0\Delta H(0)$ ($\times 10^{-4}$ T)
Si// Py	2000	0.93 ± 0.01	29.74 ± 0.07	72.8 ± 0.5	3.15 ± 0.5
	1000	0.94 ± 0.01	29.74 ± 0.07	74.7 ± 0.7	3.58 ± 0.5
	500	0.94 ± 0.01	29.77 ± 0.05	73.4 ± 0.5	3.47 ± 0.4
	200	0.94 ± 0.01	29.72 ± 0.06	75.4 ± 0.8	3.59 ± 0.5
	100	0.94 ± 0.01	29.74 ± 0.05	76.3 ± 0.9	3.64 ± 0.6
	50	0.96 ± 0.01	29.45 ± 0.11	74.3 ± 2.1	4.8 ± 1.5
	20 (fringed)	0.94 ± 0.01	29.64 ± 0.10	76.6 ± 0.5	5.25 ± 0.4
Si// Py/Pt	2000	0.94 ± 0.01	29.59 ± 0.08	109.4 ± 1.0	2.7 ± 0.7
	1000	0.94 ± 0.01	29.65 ± 0.05	106.3 ± 1.0	3.87 ± 1
	500	0.96 ± 0.01	29.22 ± 0.08	106.6 ± 0.5	2.98 ± 0.7
	200	0.94 ± 0.01	29.70 ± 0.08	107.9 ± 1.0	3.92 ± 0.8
	100	0.93 ± 0.01	29.75 ± 0.10	105.6 ± 2.0	4.91 ± 1.3
	50	0.92 ± 0.01	29.80 ± 0.20	98.3 ± 2.5	8.05 ± 1.7
	20 (fringed)	0.93 ± 0.01	29.68 ± 0.10	109.8 ± 0.2	5.04 ± 0.2
Si// Pt/Py	2000	0.91 ± 0.01	29.67 ± 0.05	100.8 ± 0.7	2.73 ± 0.4
	1000	0.92 ± 0.01	29.58 ± 0.05	100.2 ± 0.5	2.92 ± 0.3
	500	0.91 ± 0.01	29.60 ± 0.06	101.0 ± 0.7	2.92 ± 0.5
	200	0.90 ± 0.01	29.86 ± 0.07	99.2 ± 2.4	4.46 ± 1.6
	100	0.91 ± 0.01	29.70 ± 0.09	103.2 ± 1.7	3.21 ± 1.1
	50	0.91± 0.01	29.63 ± 0.10	101.0 ± 1.7	3.69 ± 1.2
	20 (fringed)	0.91 ± 0.01	29.70 ± 0.06	104.1 ± 0.5	5.11 ± 0.30

Voltage measurements of single stripe and fringed patterned samples: a comparison

To prove that the voltage signal measured in a sample with multiple parallel stripes (fringed) and that measured in a single stripe are fully equivalent, a fringed Si// Py/Pt sample with multiple parallel stripes of 50 μm each was prepared (se Fig. S1 below). The main advantage of using a fringed pattern is that the FMR absorption curve is larger than in the single-stripe case, in which the FMR absorption curve is noisy (strong improvement of the signal-to-noise ratio). Therefore, errors in the determination of ΔH and H_{res} from FMR measurements are strongly reduced. Both samples, a single-stripe sample of 5 mm × 50 μm and a fringed patterned one consisting of 41 stripes and 2 mm × 50 μm each, are sketched in Figure S1.

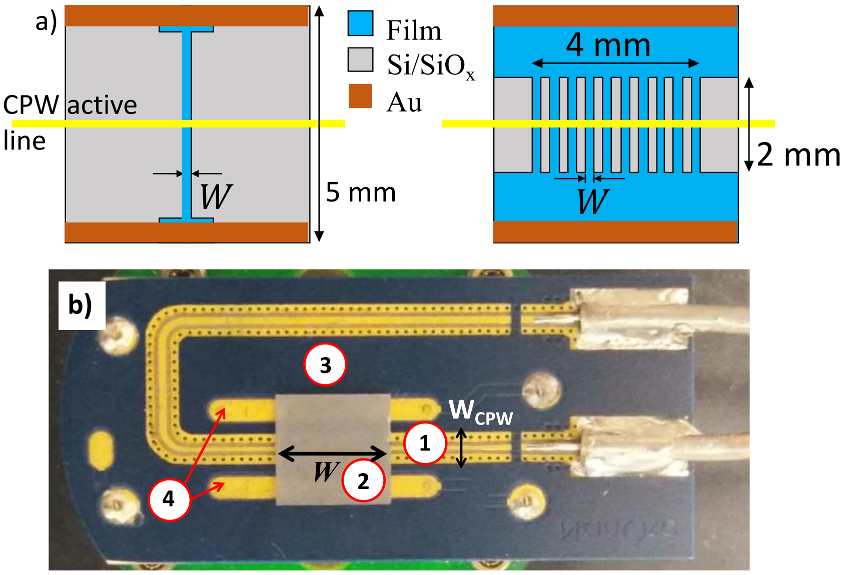

Figure. S1. *a) Schematic diagram of the experimental setup and dimensions for the single stripe samples (left) and the fringed patterned ones (right). The coplanar waveguide (CPW) (yellow line) is always transverse to the detection direction, which connects the Au contacts at both sides of the sample (brown stripes). b) Top view photograph of the actual measurement system. The numbers correspond to the following parts: (1) CPW with a total width W_{CPW} = 1.4 mm (including the RF signal active line in the middle and the two ground lines on both sides). (2) The sample placed upside down on top of the CPW with a total width of 5 mm. (3) Insulating layer. (4) Contact pads with bumps raised 20 μm above the insulating layer for making electrical contact with the sample by means of Au contacts. Finally, Kapton tape is used to fix and tighten the sample onto the CPW (not shown). This sample holder is the "CPW PPMS IP ISHE" model produced by NanOsc Instruments (http://www.nanosc.se/wave-guides.html).*

Each sample was measured as stated in the main text, with the CPW signal line running perpendicular to the stripes, and the voltage signal measured on both sides. FMR absorption curves are shown in Fig. S2. As evidenced by the Fig. S2, the FMR absorption in the fringed patterned case is nearly 40-folds larger than that in the single stripe one. This difference is attributed to the fact that the FMR absorption amplitude is proportional to the quantity of material that is being excited by the CPW underneath. As a consequence, values of ΔH and

H_{res} determined by fitting the experimental curves are much more precise in the fringed patterned sample.

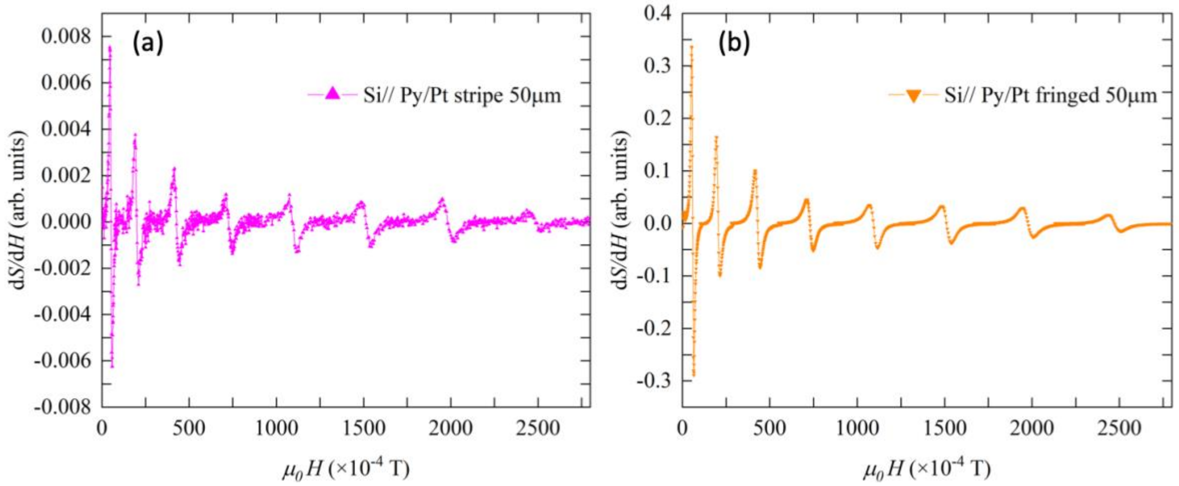

Figure S2. *Room temperature FMR absorption curves for each frequency as a function of the applied magnetic field for the single stripe sample (a), and the fringed one (b). The difference in magnitude between each other is almost 40-folds higher for the fringed patterned one, thus allowing a much more accurate evaluation of* ΔH *and* H_{res}*.*

To check that both samples are equivalent and that the patterning process has not affected their magnetic properties, FMR curves from Fig. S2 were fitted to extract ΔH and H_{res} (see **Fig. S3**) and the Kittel equations (**Eq. 2** and **Eq. 3** in the main text) were used to determine the gyromagnetic ratio (γ), the saturation magnetization (M_s), and Gilbert damping (α). These parameters are summarized in **Table S1.**

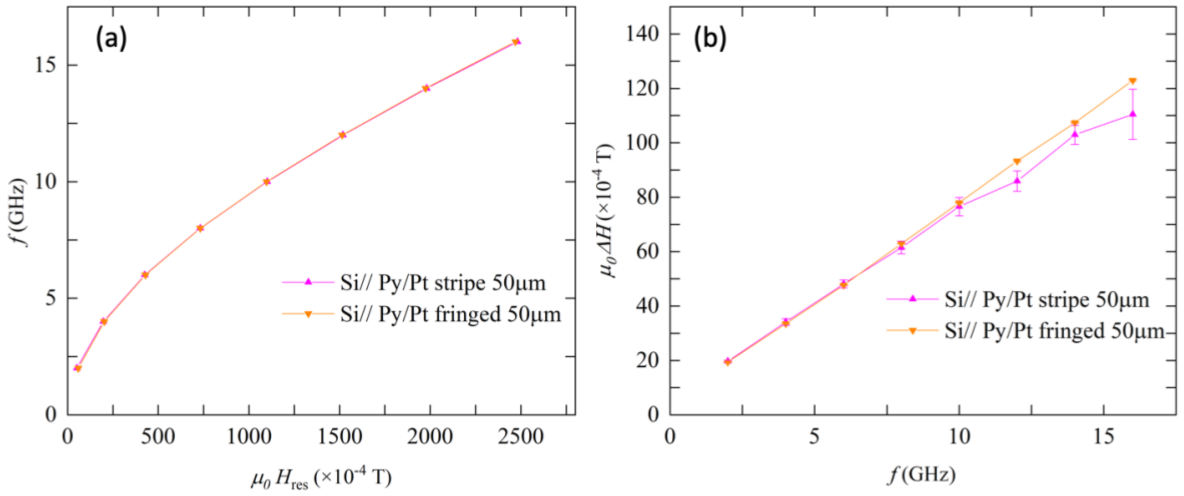

Figure S3*. FMR linewidth dependence with respect to the frequency (a), and the frequency dependence with respect to the resonance field (b). Curves for both samples show a significant overlap, thus indicating that the magnetic properties of both samples are very much alike. (Room temperature)*

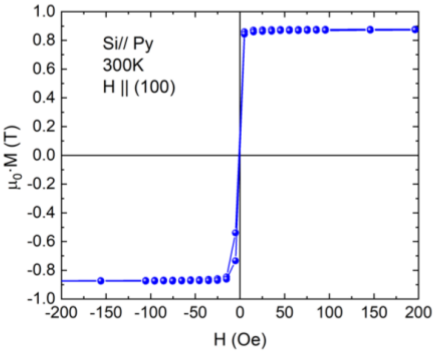

Figure S4: *Typical hysteresis loop at room temperature of one of the Py films used in this work.*

Table S3. Magnetic parameters for the fringed and stripe samples in Fig. S3 extracted by using Eq. 2 and Eq. 3 from the main text.

**Sample**	$\mu_0 M_s$ (T)	$\gamma/2\pi$ (GHz/T)	$\mu_0 H_k$ (mT)	$\alpha \times 10^{-4}$	$\mu_0 \Delta H(0)$ (mT)
Stripe	0.92	29.80	-0.31	98.3	0.80
Fringed	0.94	29.69	-0.11	110.1	0.38

From **Fig. S3** and values of magnetic parameters compiled in **Table SI**, it is evident that both samples are magnetically equivalent. The small non-linear deviation at high frequencies in **Fig. S3(a)** for the single stripe sample is attributed to the noise in the FMR curves at high frequencies which introduces a larger error in the determination of both ΔH and H_{res}.

The transversal voltage signal measured for each type of sample is depicted in **Fig. S5(a)**. The figure evidences that the two voltage curves completely overlap, having the same linewidth, resonance field, and voltage amplitude. This can be explained by the fact that the voltage in the fringed patterned sample is the average voltage over the sample's individual stripes. Additionally, it is also observed that the voltage signal does not depend on the length of the strip in the measurement direction since the active line of the CPW is only few hundred microns wide (the length of the single stripe is 5 mm, while the multiple stripes from the fringed patterned sample is 2 mm long, see **Fig. S1**).

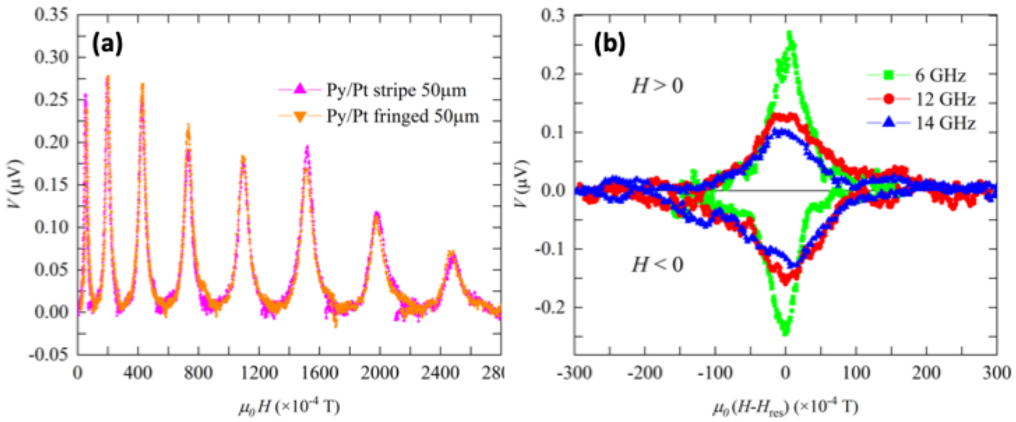

Figure S5. *a) Transversal voltage signal measured in the single stripe sample and the fringed one at room temperature. It is evident from the figure that both voltage curves completely overlap with each other, sharing linewidth, resonance field, and voltage amplitude. b) Transverse voltage measurements at three different frequencies in a Si// Py/Pt fringed patterned sample with W = 20 µm stripes, where the dominant contribution to the voltage curve should be ISHE. (Room temperature)*

Field inversion symmetry in Si// Py/Pt fringed patterned samples with *W* = 20 µm stripes

The inverse spin Hall effect (ISHE) voltage signal has to be symmetric with respect to the field direction, since it is generated by the charge current $\boldsymbol{J}_c$ that obeys the following relation

$$\boldsymbol{J}_c = \frac{2q_e}{\hbar}\theta_{SH}\boldsymbol{J}_s \times \boldsymbol{\sigma}$$

being $\boldsymbol{J}_S$ the spin current, $\hbar$ the reduced Planck's constant, q_e the electron charge, θ_{SH} the spin Hall angle and $\boldsymbol{\sigma}$ the spin polarization vector that, in our system, points to the direction of the magnetization and, therefore, towards the direction of the externally applied magnetic field. As a consequence, since $\boldsymbol{J}_s$ points always to the same direction (from the Py to the Pt), the inversion of the external DC magnetic field direction should result in the inversion of the voltage signal.

In order to prove this, we measured the transverse voltage signal applying a positive and negative magnetic field at three arbitrary frequencies in a sample of Si// Py/Pt with a fringed pattern composed of *W* = 20 µm stripes. We chose this sample because its dominant voltage contribution corresponds to ISHE. The experimental results are shown in **Fig. S5(b)**. The three voltage curves are opposite to each other, satisfying the expected symmetry relationship for an ISHE voltage signal under the inversion of the DC field.

Numerical solution of Maxwell's and LLG's equations in Fourier space.

In this appendix, we summarize our implementation of the methodology developed in Ref.**Error! Bookmark not defined.**. The two main approximations for the method are that the system is infinitely large along the in-plane directions (x and z according to the set of axes displayed in Fig. S6(a)) and that the CPW has no resistance. These two assumptions imply a translational invariance along z and reduce the problem to two dimensions.

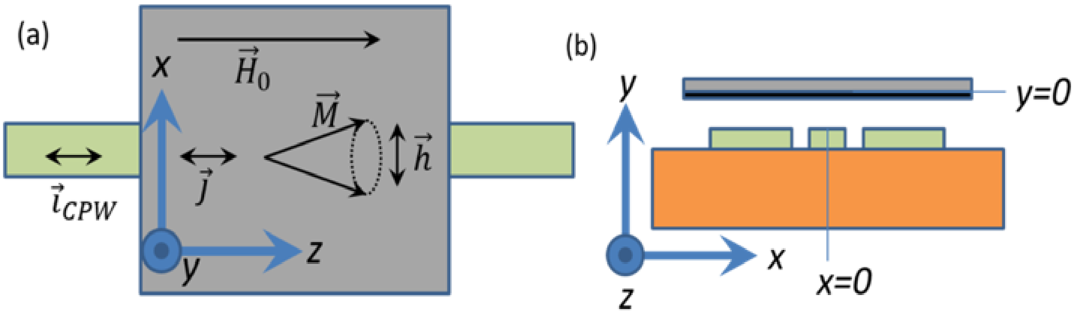

***Figure S6**: (a) Top and (b) side schematic views of the system. CPW is presented in green, and its substrate in orange, sample is in grey (Py) and black (Pt). z-axis points in the direction of the applied field along the CPW, y-axis points perpendicular to the film and outside the CPW.*

The DC magnetic field is applied along z-direction $\vec{H}_0 = H_0\hat{k}$, and thus the DC component of the magnetization is $\vec{M}_0 = M_S\hat{k}$. Oscillating parts have only components in the other two directions: $\vec{h} = h_x\hat{\imath} + h_y\hat{\jmath}$, $\vec{m} = m_x\hat{\imath} + m_y\hat{\jmath}$. As a consequence of these two facts [$h_x(x,y)$, $h_y(x,y)$, h_z=0]. Moreover, as we take the approximation $\vec{\nabla} \times \vec{h} = \sigma\vec{e}$, the only component of $\vec{e}$ different from zero is e_z.

The effective magnetic field appearing in the LLG equation:

$$\frac{\partial\vec{M}}{\partial t} = -\mu_0\gamma\vec{M} \times \vec{H}_{eff} + \frac{\alpha}{M_s}\left(\vec{M} \times \frac{\partial\vec{M}}{\partial t}\right) \quad (1)$$

is considered to include the exchange field h_{ex} but not explicitly the demagnetizing field. A demagnetizing field is included implicitly when boundary conditions are introduced. The exchange field can be accounted by the expression[1,2,3]:

$$\vec{h}_{ex} = \alpha_{ex}\nabla^2\vec{m} = \alpha_{ex}\left(\frac{\partial^2}{\partial x^2} + \frac{\partial^2}{\partial y^2}\right)\vec{m} \quad (2)$$

where α_{ex} is the exchange coupling constant. In order to numerically solve the set of equations we apply the Fourier transform along x-direction, and work with the Fourier components: $\left(\vec{m}_k, \vec{h}_k, \vec{e}_k\right) = \frac{1}{2\pi}\int_{-\infty}^{\infty} dx\left(\vec{m}, \vec{h}, \vec{e}\right)\exp(jkx)$, and consequently $\left(\vec{m}, \vec{h}, \vec{e}\right) = \int_{-\infty}^{\infty} dk\left(\vec{m}_k, \vec{h}_k, \vec{e}_k\right)\exp(-jkx)$. As $\left(\vec{m}, \vec{h}, \vec{e}\right)$ depend on y, so do $\left(\vec{m}_k, \vec{h}_k, \vec{e}_k\right)$, and the y dependence is assumed to be of exponential type $\left(\vec{m}_k, \vec{h}_k, \vec{e}_k\right) \propto \exp(qy)$ (e.g. $m_{xk}(y)$ is of the type m_{xk} $\exp(qy)$ with no further dependence of m_{xk} on y). With this, LLG equation drives to a susceptibility type relationship between the Fourier components of magnetization and magnetic field (by disregarding any non-linear term in $\vec{h}_k$ or $\vec{m}_k$):

$$\vec{m}_k = \begin{pmatrix} \chi_{xx} & j\chi_{xy} \\ -j\chi_{xy} & \chi_{yy} \end{pmatrix}\vec{h}_k \quad (3)$$

where the susceptibility components are given by:

$$\chi_{xx} = \chi_{yy} = \frac{-j\gamma' M_s[\alpha - j\gamma' H_0 + j\alpha_{ex}\gamma' M_s(-k^2+q^2)]}{1 + [\alpha - j\gamma' H_0 + j\alpha_{ex}\gamma' M_s(-k^2+q^2)]^2}$$
$$\chi_{xy} = \frac{-\gamma' M_s}{1 + [\alpha - j\gamma' H_0 + j\alpha_{ex}\gamma' M_s(-k^2+q^2)]^2} \quad (4)$$

where $\gamma' = \frac{\gamma}{\omega}$. With this, the combination of Maxwell's and LLG equations drives to:

$$qe_{zk} = -j\omega\mu_0\left[h_{xk}(1 + \chi_{xx}) + jh_{yk}\chi_{xy}\right] \quad (5)$$
$$jke_{zk} = -j\omega\mu_0\left[h_{yk}\left(1 + \chi_{yy}\right) - jh_{xk}\chi_{xy}\right] \quad (6)$$
$$\sigma e_{zk} = -jkh_{yk} - qh_{xk} \quad (7)$$
$$0 = -jk\left[h_{xk}(1 + \chi_{xx}) + jh_{yk}\chi_{xy}\right] + q\left[h_{yk}\left(1 + \chi_{yy}\right) - jh_{xk}\chi_{xy}\right] \quad (8)$$

It can be easily seen that (8) can be obtained from (5) and (6). Thus, we end up with three homogenous equations with three unknowns that will present solutions different from zero only if the determinant of the system matrix is zero. This drives to the dispersion relation relating q and k:

$$(1+\chi_{xx})(q^2-k^2)-j\delta^{-2}\left[(1+\chi_{xx})^2-\chi_{xy}^2\right]=0 \tag{9}$$

where δ is classical skin depth $(\sigma\omega\mu_0)^{1/2}$. Equations (3) and (5)-(7) allow expressing, as a function of e.g. h_{xk}, all the other Fourier coefficients (h_{yk}, e_{zk}, m_{xk}, and m_{yk}). Introducing expressions (4), (9) becomes a polynomial of order 3 in q^2. Thus, in the magnetic part (i.e. permalloy), for every k there are six possible values of q and the general solution of h_{xk} is a linear combination of the six exponential terms. In the non-magnetic part of the system, where the susceptibility tensor can be taken as 0, relation (9) renders two values of q for every k and the general solution will only contain two terms. In total, for every value of k, the problem has eight coefficients that are determined by imposing the boundary conditions specified in the boundary conditions at the end of this section.

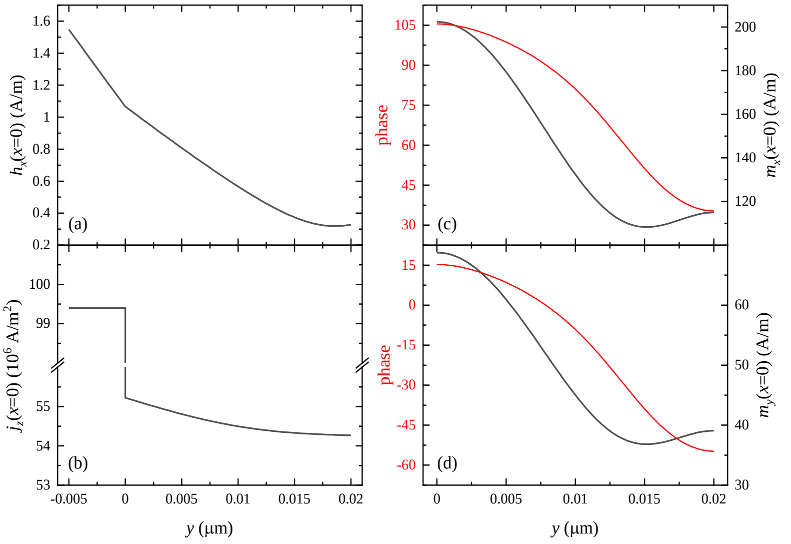

Figure S7: *Calculated variation across the sample (at the center x=0) of: (a) modulus of the x component of magnetic field, (b) current density, and (c and d) magnetization components, phase (in red left axis) and modulus (black, right axis). Parameters used for the calculations are: s= 20 µm; W_{CPW}=400 µm (width of the CPW); Δ= 400 µm (gap width of the CPW); d= 500 µm; f = 10GHz; γ= 28.9 GHz/T; μ_0H_0= 0.11756 T; μ_0M_S= 0.9 T (M_S=7.16 10^5 A/m); α = 0.01; δ_{Pt}= 1.1239 µm; δ_{Py}= 1.5078 µm; t_{Pt}= 5 nm; t_{Py}= 20 nm; α_{ex}=6.3 10^{-8} $(\mu m)^{-2}$ intensity by the CPW= 1 mA. The region with y<0 (>0) corresponds to Pt (Py). Applied field is close to calculated resonance.*

Once the eight values of q and the eight coefficients are determined for every value of k, Fourier terms at every point y can be calculated and the inverse Fourier transform numerically done. As an example, Fig. S7 shows the dependence on y of some quantities calculated at the center of the CPW (x=0).

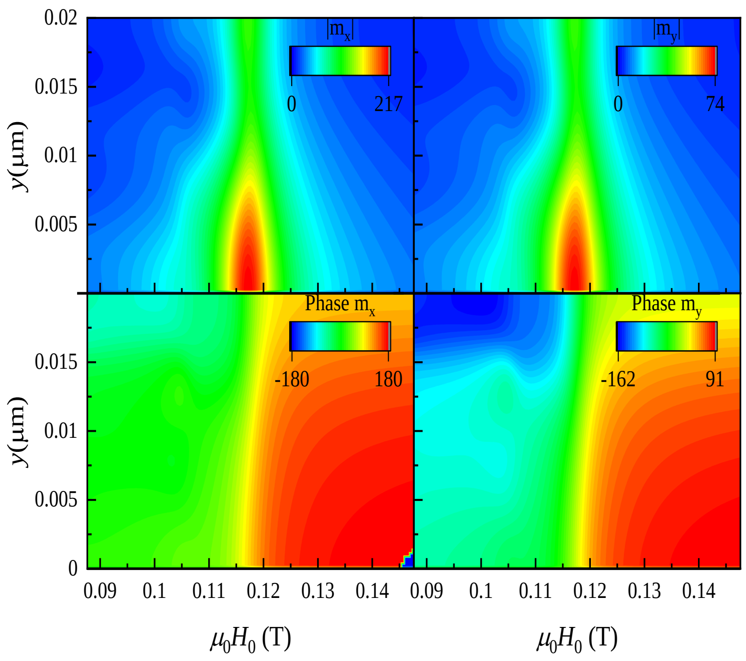

Figure S8: *Modulus (in A/m) and phase (in degrees) of m_x and m_y across the film (at x=0) when changing the applied field across resonance. All parameters (but H_0) are the same that in figure S7.*

By varying the value of the applied magnetic field, the resonance can be appreciated as a maximum oscillation of m_x and m_y when applied magnetic field reaches the resonance value, as depicted in Fig. S8.

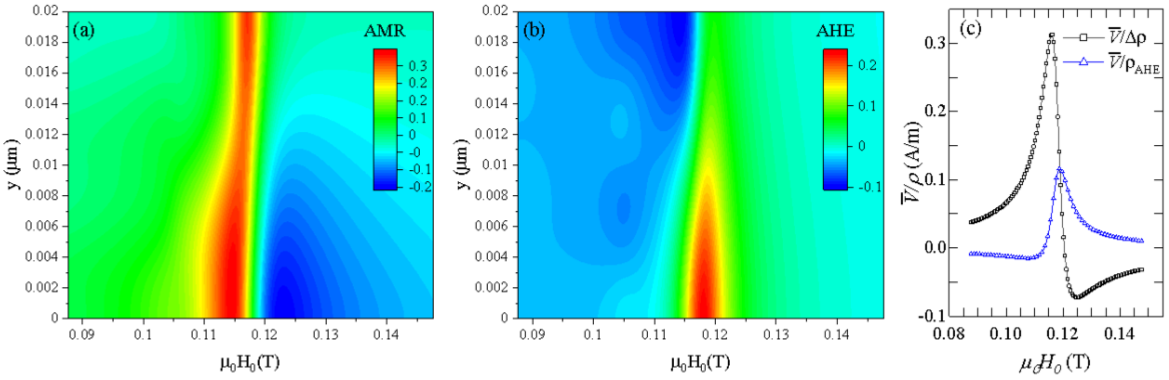

Figure S9: *Rectification voltage calculated by expression (13), (a) plots the expected contribution to V of AMR (divided by Δρ in A/m) and (b) that from AHE (divided by ρ_{AHE} in A/m). Panel (c) plots the same quantities averaged over the film thickness. All parameters used for the calculations, except H_0, are the same that in Fig. S7. It is worth noting that the sign convention in this figure is the opposite to that in Figs. 2a, 3a, 4a, and 5a in the main text.*

In order to calculate the SRE effect, one must recall the generalized Ohm's law

$$\vec{e} = \rho_{\perp}\vec{j} + \frac{\Delta\rho}{M_s^2}(\vec{j}\cdot\vec{M})\vec{M} - \frac{\rho_{AHE}}{M_s}(\vec{j}\times\vec{M}) \qquad (10)$$

The contributions along x direction in these expressions are:

$$e_x = \frac{\Delta\rho}{M_s} j_z m_x + \frac{\rho_{AHE}}{M_s} j_z m_y \tag{11}$$

where the quantities here include the time dependent factor $exp.(j\omega t)$. The DC component of this field will be given by the real part ($\Re$) of its time average. It can be easily shown that:

$$\Re\langle e_x\rangle = \frac{1}{2}\frac{\Delta\rho}{M_s}\Re(j_z m_x^*) + \frac{1}{2}\frac{\rho_{AHE}}{M_s}\Re\left(j_z m_y^*\right) \tag{12}$$

where in the RHS of eq. 12, quantities refer to the pre-factor accompanying the time dependence factor $exp.(j\omega t)$. The SRE voltage is then given by $V_{SRE} = -\int_{-\infty}^{\infty}\Re\langle e_x\rangle dx$, thus:

$$V_{SRE} = -\frac{1}{2}\frac{\Delta\rho}{M_s}\int_{-\infty}^{\infty}\Re(j_z m_x^*)dx - \frac{\rho_{AHE}}{M_s}\int_{-\infty}^{\infty}\Re\left(j_z m_y^*\right)dx \tag{13}$$

Last integrals can be easily computed in Fourier space using the Parseval's identity:

$\int_{-\infty}^{\infty}\Re(j_z m_i^*)dx = 2\pi\Re\left(\int_{-\infty}^{\infty} j_{zk} m_{ik}^* dk\right)$ (i= x,y). Thus:

$$V_{SRE} = -\pi\frac{\Delta\rho}{M_s}\Re\left(\int_{-\infty}^{\infty} j_{kz} m_{kx}^* dk\right) - \pi\frac{\rho_{AHE}}{M_s}\Re\left(\int_{-\infty}^{\infty} j_{kz} m_{ky}^* dk\right) \tag{14}$$

where the first and second terms in RHS of eq. 14 give the contribution to SRE of AMR and AHE respectively. The factors accompanying the parameters $\Delta\rho$ and ρ_{AHE} are plotted in Fig. S9. As j_{kz} and m_{kx}-m_{ky} depend on y, so it does the rectification voltage created, as illustrated by Figs. S9(a) and (b). The average (along y) is plotted in Fig. S9(c). This figure shows that factors accompanying AMR and AHE terms (labeled as $V/\Delta\rho$ and V/ρ_{AHE}) are of the same order of magnitude. Considering that for permalloy, $\Delta\rho/\rho$ is about 4% at room temperature[4] and ρ_{AHE}/ρ is at least one order of magnitude smaller,[5] we can conclude that the dominant term in the SRE is the AMR and that AHE one can be disregarded.

At this point it is of major relevance to highlight that the coordinate system in Fig. S6 has the transversal axis x in the opposite direction to our experimental setup. As a consequence, the sign of the voltage is changed between figure S9 and our experimental setting (Figs. 2a, 3a, 4a, and 5a). To evidence this, it is enough to consider the expected sign of the ISHE voltage according to coordinates in Fig. S6, set as the positive direction in our experimental setup. This voltage appears because of the electric field that compensates the intensity appearing in Pt induced by the spin current. This intensity**Error! Bookmark not defined.** is proportional to $\hat{n}\times\hat{s}$ where $\hat{n}$ is the direction of propagation of the spin current, (0,-1,0) in our case, and $\hat{s}$ is the direction of the spin injected, (0,0,1) in our case. Thus $\hat{n}\times\hat{s}$ = (-1,0,0) and the induced current runs towards negative x. The electric field (e_{ISHE}) appearing to compensate this current is then pointing towards positive x this meaning that the voltage drops $V_{\mathrm{ISHE}} = -\int_{-\infty}^{\infty} dx e_{\mathrm{ISHE}} < 0$.

Introduction of finite size along *z*:

In order to evaluate the effect of the finite size of the sample along the direction of the CPW, we assume that the main effect of the walls at the end of the sample is the accumulation of charge on them due to the current created inside the sample. Moreover, we treat this charge accumulation as a perturbation of the exact solution presented above, so $\vec{e} = \vec{e}^{\infty} - \Delta\vec{e}$ where $\vec{e}^{\infty}$ is the field found for an infinite sample and $\Delta\vec{e}$ is the field created by the charge accumulation that, for simplicity is assumed to be along *z* direction. The charge accumulated at the ends of the sample is described as a linear charge density, as the size of the length of the system along the CPW is always much larger than the thickness of the sample. In general, the *z* component of the field created at a point (x,0,z) by an arbitrary linear charge density $\lambda(x)$ placed along x at a z=0 is:

$$e_{\lambda z}(x,0,z) = \frac{z}{4\pi\epsilon_0}\int_{-\infty}^{\infty} d\xi \frac{\lambda(\xi)}{[(x-\xi)^2+z^2]^{3/2}} \qquad (15)$$

And in Fourier space, the components of this field will be given by the modified Bessel function of 2nd kind:

$$e_{\lambda kz}(k,0,z) = -\lambda_k \frac{|k|}{2\pi\epsilon_0} K_1(|kz|) \qquad (16)$$

where $\lambda_k = \frac{1}{2\pi}\int_{-\infty}^{\infty} dx\lambda(x)\exp(jkx)$ are the Fourier components of the linear charge density.

If the size of our system along z is W we can consider that there are two linear distributions of charge placed at $z = \pm W/2$ with opposite charges. Thus, the Fourier components of Δe_z are given by:

$$\begin{aligned}\Delta e_{kz}(k,0,z) &= -\lambda_k \frac{|k|}{2\pi\epsilon_0}\left[K_1\left(\left|k\left(\frac{W}{2}-z\right)\right|\right) - K_1\left(\left|k\left(z-\frac{W}{2}\right)\right|\right)\right] \\ &\equiv -\frac{\lambda_k}{2\pi\epsilon_0}\Delta_{Wk}\end{aligned} \qquad (17)$$

Then, the current density will be given by $j_z = j_z^{\infty} + \sigma\Delta e_z$. Moreover, as the lineal intensity is generated by the current arriving to the end of the sample, it will be given by $\lambda = \int_0^t d\tau \int_{-t_{Pt}}^{t_{Py}} dy j_z(y)$, or in Fourier space by $\lambda_k = \int_0^t d\tau \int_{-t_{Pt}}^{t_{Py}} dy j_{zk}(y)$. The integral along y must be divided into the two materials:

$$\begin{aligned}\int_{-t_{Pt}}^{0} dy j_{kz} &= \int_{-t_{Pt}}^{0} dy j_{kz}^{\infty} + \sigma_{Pt} t_{Pt} \Delta e_{kz} \\ \int_{0}^{t_{Py}} dy j_{kz} &= \int_{0}^{t_{Py}} dy j_{kz}^{\infty} + \sigma_{Py} t_{Py} \Delta e_{kz}\end{aligned} \qquad (18)$$

To simplify notation, we define $J_{kPt} = \int_{-t_{Pt}}^{0} dy j_{kz}$, $J_{kPt}^{\infty} = \int_{-t_{Pt}}^{0} dy j_{kz}^{\infty}$, $J_{kPy} = \int_{0}^{t_{Py}} dy j_{kz}$ and $J_{Py}^{\infty} = \int_{0}^{t_{Py}} dy j_{kz}^{\infty}$. With this, it can be shown that the Fourier components of the integrated current density at the permalloy as a function of size are given by:

$$J_{kPy} = \frac{J_{kPy}^{\infty}[1 - j\gamma_{Pt}\Delta_{Wk}] + jJ_{Pt}\gamma_{Pt}\Delta_{Wk}}{1 + \Delta_{Wk}(\gamma_{Py} + \gamma_{Pt})} \qquad (19)$$

where $\gamma_X \equiv \frac{\sigma_X t_X}{2\pi\epsilon_0}$ with X=Py or Pt. This expression allows to compute the average (along y and z) of rectification voltage as a function of the size of the system W, which are depicted with the sign convention used in experimental measurements (multiplied by -1 with respect to the reference system in Fig. S5) in Figure 7.

Boundary conditions:

According to Balaji & Kostilev the boundary conditions to be fulfilled are:[1]

1. At Pt surface:

$$h_{yk} \coth[|k|(d+s)] - j\frac{|k|}{k} h_{xk} = j\frac{\sinh(|k|d)}{\sinh[|k|(d+s)]}\frac{|k|}{k} j_k$$

   where d is the thickness of the CPW substrate and s the separation between the Pt surface and the CPW; j_k are the Fourier coefficients of the intensity running by the CPW. The calculation of j_k is detailed in Ref. [**Error! Bookmark not defined.**].

2. At Pt/Py interface, continuity of h_x and $b_y = h_y + m_y$ must be satisfied. This implies the continuity of their Fourier coefficients.
3. At Pt/Py interface (and at the end of Py) we neglect any surface anisotropy implying that $\frac{\partial \vec{m}}{\partial y} = 0$ at both surfaces.
4. At the end of Py, the boundary condition reads:

$$h_{yk} + m_{yk} + jh_{xk}\frac{k}{|k|} = 0$$

---

[1] S. Balaji and M. Kostylev. *A two-dimensional analytical model for the study of ferromagnetic resonance responses of single and multilayer films.* J. Appl. Phys. 121, 123906 (2017)

[2] D. Stancil and P. Prabhakar in "*Spin Waves, Theory and Applications*", Springer 2009.

[3] Exchange field has also a component proportional to $\vec{M}$ but it vanishes when taking the cross product with $\vec{M}$ in LLG equation.

[4] T.R. McGuire and R.I Potter, Anisotropic magnetoresistance in ferromagnetic 3d alloys, IEEE Trans. Magn. **11**, 1018 (1975)

[5] Y.Q. Zhang, N.Y. Sun, R. Shan, J.W. Zhang, S.M. Zhou, Z. Shi, and G.Y. Guo, Anomalous Hall effect in epitaxial permalloy thin films, J. Appl. Physics **114**, 163714 (2013).
